# Clinicopathological Profile of Pure Neuroendocrine Neoplasms of the Esophagus: A South Indian Center Experience

**DOI:** 10.1155/2016/2402417

**Published:** 2016-05-31

**Authors:** Govind Babu Kanakasetty, Loknatha Dasappa, Kuntegowdanahalli Chinnagiriyappa Lakshmaiah, Mangesh Kamath, Linu Abraham Jacob, Suresh Babu Mallekavu, Lakkavalli Krishnappa Rajeev, Rudresha Antapura Haleshappa, Lokesh Kadabur Nagendrappa, Smitha Carol Saldanha, Rekha V. Kumar

**Affiliations:** Department of Medical Oncology, Kidwai Memorial Institute of Oncology, Dr. M. H. Marigowda Road, No. 30, Bangalore 560029, India

## Abstract

*Purpose*. Neuroendocrine neoplasms (NENs) of the esophagus are very uncommon with only a few studies published worldwide. Studies on clinical profile, management, and outcomes are very uncommon.* Methods*. We report the largest single institution retrospective review of 43 patients of pure esophageal NENs out of our registry of gastrointestinal neuroendocrine tumors treated between 2005 and 2014. Data on the incidence, tumor location, clinical symptoms, stage at presentation, grading, treatment protocol, and treatment outcomes was collected and analyzed.* Results*. Among 1293 cases of esophageal cancers, pure esophageal NENs were diagnosed in 43 cases. The mean patient age was 55.8 years. The male : female ratio was 1.5 : 1. 81.4% of the tumors were located in the lower third of the esophagus and gastroesophageal junction. Neuroendocrine carcinomas (NEC; G3) accounted for the vast majority of NENs (83.7%). 53.5% patients were Stage IV and 32.5% were Stage III at presentation. The combined median survival of stages II and III patients was 18.25 months, with treatment. The median survival of treated patients with metastatic disease was 6.5 months.* Conclusion*. Esophageal NENs most commonly were neuroendocrine carcinomas, presented in metastatic stage and were associated with poor prognosis. Grade 2 (G2) tumors had better outcomes than NEC (G3). In nonmetastatic disease, presence of lymph node metastasis and unresectable disease had poorer outcomes.

## 1. Introduction

Neuroendocrine neoplasms (NENs) of the digestive system {gastroenteropancreatic (GEP)}, tumors arising in the gastrointestinal tract and the pancreas are uncommon. The annual incidence in the United States is about 3.65 per 100,000 population [1]. An increase in incidence is being seen worldwide due to increased physician awareness and better diagnostic methods. NENs in the esophagus are rare [2, 3]. Data on the epidemiology and experience of clinical features, management, and prognosis are emerging [4, 5]. Two large studies from Korea were published recently describing the epidemiology and the proposed treatment strategies. There are only a few large studies single center studies from other countries regarding experience with primary esophageal NENs. However, recent reviews have highlighted the emerging incidence of this entity. In this study, we report a series of 43 cases of pure NENs of the esophagus diagnosed between 2005 and 2015 in our regional cancer center. The incidence, clinicopathologic features, immunohistochemical findings, treatment modalities, and prognosis of primary esophageal NENs that were treated in our institute are described in this study.

## 2. Materials and Methods

A retrospective analysis of all patients diagnosed with primary esophageal NENs from 2005 to 2015 in our center was done. All pathologically confirmed NENs of the esophagus were included and their clinical data was collected. Patient age, gender, presenting symptoms, presenting Eastern Cooperative Oncology Group Performance Status (ECOG-PS), smoking, tobacco chewing and alcohol abuse habits, pathological findings including mitotic count and immunohistochemical expression of synaptophysin, chromogranin, and the Ki-67 labeling index, lymph node (LN) and visceral metastases, types of treatment, and overall survival were all recorded and analyzed.

The esophageal tumor locations were divided based on endoscopic findings into U3 (upper third; 15–25 cm from the incisor teeth), M3 (middle third; 25–30 cm from the incisor teeth), and L3-gastroesophageal junction (GEJ) (lower third; 30–40 cm from the incisor teeth). Staging workup to detect regional LN metastasis or distant metastasis was mainly evaluated using contrast enhanced computerized tomography and stage grouping was done according to the AJCC/UICC staging manual 7th Edition published in 2010. All tumors were subjected to cytokeratin immunohistochemistry (IHC). Neuroendocrine differentiation was confirmed using immunohistochemical staining for synaptophysin and chromogranin.

Grading of the NETs was done based on proliferation according to the 2010 World Health Organization (WHO) classification for digestive system neuroendocrine tumors based on the mitotic count and Ki-67 labeling index. The tumors were to be graded into three tiers (G1, G2, and G3) system according to the following definitions of mitotic count and Ki-67 index: G1: mitotic count <2 per 10 high power fields (HPF) and/or ≤2% Ki-67 index; G2: mitotic count 2–20 per HPF and/or 3–20% Ki-67 index; G3: mitotic count >20 per HPF and/or >20% Ki-67 index. Mitotic counting was done in at least 50 HPFs whenever possible in resected/biopsy specimens. The Ki-67 index using the MIB 1 antibody was calculated as a percentage of 500–2000 cells counted in areas of strongest nuclear labeling. When grade differed for mitotic count and Ki-67 index for the same tumor, the higher grade was assumed. By definition, G1 and G2 tumors were termed as neuroendocrine tumors (NETs) and G3 tumors were termed as neuroendocrine carcinomas (NECs). The details of antibodies used for immunohistochemistry are given in Table 1. All hematoxylin-eosin and IHC slides were reviewed by one of the authors (RVK) and additional immunostaining for Ki-67 antibody was performed for the older cases wherever possible. It is to be importantly noted that the whole specimen of the surgically resected cases was microscopically examined entirely such that NENs coexisting with adenocarcinoma or squamous cell carcinoma histologies were excluded. In the inoperable or unresectable cases, a minimum of 3 endoscopic random biopsies were taken from different sites of the macroscopic lesion and each biopsy tissue was carefully examined microscopically to rule out a coexisting noon-neuroendocrine component. Therefore, only pure NENs were included in this study.

Overall survival was calculated from the date of diagnosis to the date of death or last follow-up (in months). The data was compared with the global published data on esophageal neuroendocrine neoplasms.

## 3. Results

A total of 43 pure esophageal NENs were diagnosed out of 1293 cases of esophageal cancers constituting 0.03% of all the esophageal cancers presenting to our institute. The baseline characteristics of the 43 patients analyzed are shown in Table 2.

The mean age of the patients was 55.8 years. 88.4% patients were aged 40 years or more. The male to female ratio was 1.5. The most common symptom at presentation was dysphagia (*N* = 38; 88.3%). The other common symptoms were abdominal or retrosternal pain (18.6%), loss of weight, and/or loss of appetite (18.6%) and melena (4.6%). None of the patients had carcinoid syndrome. Thirty-four patients (79%) presented with ECOG-PS 1 or PS 2. Nine patients presented with ECOG-PS 3 (21%). Twelve patients (27.9%) had either the tobacco smoking or tobacco chewing habit. Thirty-five patients (81.4%) had tumor located either in lower third or in gastroesophageal junction. 83.7% patients had G1 (grade 1) tumors and 16.3% patients had G2 (grade 2) tumors; there were no patients with G3 (grade 3) tumor. All tumors were cytokeratin positive by IHC. Synaptophysin staining was positive in 97.7% and chromogranin staining was positive in 93.2% patients.

Treatment outcomes of all the patients are as outlined in Tables 2 and 3. Twenty-three patients (53.5%) presented with metastatic disease and twenty patients (46.5%) presented with locally advanced disease (stage II and stage III) comprising either regional node negative or positive subset of patients (as per AJCC/UICC staging manual 7th Edition published in 2010). There were no stage I presentations. Of the 20 patients who were locally advanced at presentation, 13 patients who were regional lymph node negative underwent upfront surgery with or without perioperative chemotherapy with the cisplatin and etoposide combination. Two patients with either node positivity or extra-tumoral extension (invasion of pleura, pericardium, or diaphragm) underwent neoadjuvant chemoradiotherapy, with cisplatin used concurrently with radiation. Patients with locally advanced disease who underwent surgery had a median survival of 18.25 months. Seven patients with locally advanced disease were unable to undergo surgery and were either treated with chemotherapy or underwent treatment with monthly long acting Octreotide injections (Octreotide LAR) based on the grade of tumor. The median overall survival of locally advanced disease was 18 months.

Of the 23 patients with stage IV disease, two were G2 NETs who underwent treatment with monthly long acting Octreotide injections. The G3 metastatic NECs were treated with palliative cisplatin and etoposide chemotherapy. The median overall survival of stage IV disease patients was 6.5 months. The median survival of patients with ECOG-PS 1/2 and PS 3 was 17.25 months and 6 months, respectively.

## 4. Discussion

This study, where we have analyzed 43 cases of pure NENs of the esophagus, is to the best of our knowledge the largest single center clinic-pathological study to date on this subject worldwide and certainly of the South East Asia region [5–20]. Esophageal NENs are reported to constitute less than 1% of all esophageal cancers [16]. Several smaller studies have reported incidence of NENs to be 0.05–7.6% of all esophageal cancers [5–7, 11–13, 16–18, 20]. In our study, 0.03% of all esophageal cancers were esophageal pure NENs. Our study confirms the rarity of esophageal NENs; the rarity of this malignancy has meant that there are no validated protocols for the management of this rare disease [7–15]. The increasing incidence of esophageal NENs has been postulated to be due to a better understanding of this disease entity and its recognition due to the newer, simple technique of immunohistochemistry. It is important to note that in our study esophageal cancers composed of mixed neuroendocrine and adenocarcinoma/squamous cell carcinoma were excluded; only pure NENs were analyzed. In a large review on GEP-NENs by Ilett et al., it was noted that many of the cases of esophageal NENs were “mixed NENs” composed of the neuroendocrine and nonneuroendocrine (adenocarcinoma or squamous cell carcinoma) component [17]. In the study by Maru et al., mixed NECs together with the epithelial component were found in sixteen out of forty patients; pure NECs were diagnosed in the remaining twenty-four patients only [12].

The comparison between features of our study and other similar studies is highlighted in Table 4.

The mean age of the patients in our study was 55.8 years which is lower than previous published studies [5, 7, 12, 15–18, 20]. The male : female ratio in our study is 1.5 which is lower than the recent large studies. Similar to all the other studies, dysphagia was the most common presenting symptom. The proportion of smokers and tobacco chewers in our study was 27.9%; in the study by Ku et al., 41% of patients were smokers [15]. The proportion of esophageal NENs occurring in the lower esophagus was 81.4%; this finding is similar across all the large studies and parallels the increase in endocrine cell number in this part of the esophagus. 83.7% of the patients had G3 disease, which confirms the finding across all the recent reports that NENs of the esophagus are predominantly high grade. The proportion of stages III, IV disease in our study was 86%; this is the largest proportion of advanced/metastatic disease when compared to all the previous studies.

In our center, we practice a protocol wherein esophageal NENs undergo surgery upfront if resectable or neoadjuvant chemoradiotherapy followed by surgery if borderline is resectable. Chemotherapy with cisplatin and etoposide regimen is given either in the adjuvant setting or with palliative intent in unresected or metastatic NECs. The G1 or G2 tumors which are not completely resected or metastatic are treated with long acting somatostatin analogues. Whereas there is no consensus on the guidelines for the management of esophageal NENs due to their rarity, our management protocol appears to be similar to that mentioned in the studies by Lee et al. and Tao et al. [7, 19].

The median survival of nonmetastatic disease was 18.25 months; the survival in this subset was improved with surgery and perioperative chemotherapy or neoadjuvant chemoradiotherapy. In the locally advanced group, lymph node positivity and unresected disease status were associated with the poorest outcome in the nonmetastatic group. The median survival of the lymph node negative group was 43 months and the median survival of node positive/stage III disease was 13.5 months. In the study by Lee et al., lymph node positivity was noted to be of prognostic value, as affirmed in our study [7]. The median survival of patients with metastatic disease was 6.5 months with better outcome noted in the two patients with G2 (grade 2) disease treated with Octreotide LAR (16 months). This confirms the fact that, for a presenting stage of disease, higher histological grade adversely impacts the outcome. In our experience, chemotherapy has shown a benefit not only in the overall survival but also in improving the performance status; the benefit is however short-lived. In a recently published meta-analysis, it was concluded that chemotherapy improved the overall survival of small cell carcinoma of esophagus (G3) [20]. Many of the previous studies included mixed NENs of the esophagus. In a study, mixed NENs were noted to have better outcomes than pure NENs [14]. Our study suggests that the outcomes of pure NENs are comparable to that of mixed NENs which are as good for pure NENs also. This observation needs to be confirmed in future studies.

## 5. Conclusion

To the best of our knowledge, this is the largest study worldwide and certainly of the South East Asia region on the clinical profiles and outcomes of esophageal pure neuroendocrine neoplasms. Although the general incidence of gastrointestinal neuroendocrine cancers is increasing, the incidence of esophageal NENs is still very rare. In our study, esophageal NENs most commonly presented in a metastatic stage were G3 NECs and associated with poor prognosis. G2 NETs had a better outcome than G3 NECs and lymph node metastasis and unresectable disease had poorer outcomes. Whenever feasible, surgery is the treatment of choice, conferring a significant survival advantage. In metastatic disease, chemotherapy or somatostatin analogues as per tumor grade help in symptom palliation with modest survival rates. Many of the previous studies included tumors with mixed neuroendocrine and epithelial component. Therefore, larger studies are required to determine optimal management of pure esophageal neuroendocrine neoplasms.

## Figures and Tables

**Table 1:** Details of IHC antibodies.

Serial number	Antibody	Clone	Dilution	Source
1	Ki-67/MIB 1	MIB 1	1 : 70	Biogenex
2	Synaptophysin	SNP 88	1 : 80	Biogenex
3	Chromogranin	LK2H10	1 : 300	Biogenex
4	Cytokeratin	C 11	1 : 80	Biogenex

**Table 2:** Baseline characteristics of esophageal NENs.

Variable	Number (percentage)
*Age*	
<40 years	5 (11.6%)
40–60 years	15 (34.9%)
>60 years	23 (53.5%)

*Gender*	
Male	26 (60.5%)
Female	17 (39.5%)

*Symptoms*	
*Dysphagia*	38 (88.3%)
Abdominal/retrosternal pain	9 (22.5%)
Loss of weight or loss of appetite	8 (18.6%)
Gastrointestinal bleed	2 (4.6%)
Carcinoid syndrome	0

*Performance status*	
1/2	34 (79%)
3	9 (21%)

*Esophagus tumor location*	
Upper third (15–25 cm from incisor)	1 (2.3%)
Middle third (25–30 cm from incisor)	7 (16.3%)
Lower third, GE junction (30–40 cm from incisor)	35 (81.4%)

*Mitotic count and Ki-67 index (WHO, [21])*	
G1: <2/10 HPF's; <2%	0
G2: 2–20/10 HPF's; 3–20%	7 (16.3%)
G3: >20/10 HPF's; >20%	36 (83.7%)

*Disease stage*	
I	0
II	6 (14%)
III	14 (32.5%)
IV	23 (53.5%)

*Treatment received*	
*STAGE II, III (n* = 20; 46.5%)	*mOS = 18.25 m (2.5–60 m) n* = 13
Upfront surgery (THE/TTE) +/− CT	*mOS = 18.5 m (2.5–60 m) n* = 2
NACT plus surgery	*mOs = 15.25 m*
Received Chemo/Oct LAR only	*n* = 7* mOS = 12 m (6–30 m)*
*STAGE IV (n* = 23; 53.5%)	*mOS = 6.5 (1.5–32 m)*
Cisplatin + etoposide	*n* = 21* mOS = 6.5 m (1.5–32 m)*
Octreotide LAR	*n* = 2* mOS = 16 m*

*mOS stage II (n* = 6)	*43 m (18–60 m)*

*mOS stage III (n* = 14)	*12.5 m (6–30 m)*

**Table 3:** Treatment received and outcomes.

Stage	Number of patients (percentage)	Median overall survival (mOS)	Range of survival
*Stage I*	*n* = 0	0	0

*Stage II*	*n* = 6	43 m	18–60 m

*Stage III*	*n* = 14	12.5 m	6–30 m

*Stage II *and* stage III*	*n* = 20; (46.5%)		
(1) Upfront surgery	*n* = 12	19.75 m	6–60 m
(THE/TTE +/− Cth)			
(2) NACT + surgery	*n* = 2	16 m	14–18 m
(3) Cth/LAR; no Surgery	*n* = 6	8.75 m	6–13 m

*Stage IV*	*n* = 23; (53.5%)		
(1) Cisplatin + etoposide (G3)	*n* = 21	6.5 m	1.5–32 m
(2) Octreotide LAR (G2)	*n* = 2	16 m	

**Table 4:** Comparison of studies on esophageal NENs.

Study^*∗*^	Number of cases	Mean age (years)	Gender ratio	% of grade 3	Lower 1/3rd, GE Jn location	Stage III, IV proportion	Median OS (months)
Yun et al. [5]	21	56	3.2 : 1	100%	38%	61.9%	18.3
Lee et al. [7]	26	60	4 : 1	38.5%	76.9%	57.7%	27
Maru et al. [12]	40	60	7 : 1	100%	82.5%	45%	14
Ku et al. [15]	22	60	4.5 : 1	100%	86%	36%	19.8
Pantvaidya et al. [16]	18	62	2.6 : 1	100%	17%	61%	6
Our study Govind Babu^*∗*^ et al. (2015)	43	54.8	1.5 : 1	83.7%	81.4%	86%	12

^*∗*^Comparison made only for studies with more than 15 cases.
